# Spatial distribution and influencing factors analysis of national key rural tourism villages in the Yangtze River Delta region based on geographically weighted regression

**DOI:** 10.1371/journal.pone.0291614

**Published:** 2023-11-15

**Authors:** Haiyong Zhao, Junnan Zheng, Sihui Ma, Lei Zhao, Pengfei Xu, Jian Li

**Affiliations:** 1 College of Landscape Architecture, Zhejiang Agriculture and Forestry University, Hangzhou, China; 2 Zhejiang Informatization Development Institute, Hangzhou Dianzi University, Hangzhou, China; 3 School of Management, Zhejiang University of Technology, Hangzhou, China; 4 Zhejiang Province Key Think Tank: Institute of Ecological Civilization, Zhejiang A&F University, Hangzhou, China; Northeastern University (Shenyang China), CHINA

## Abstract

National key rural tourism villages (NKRTVs) can lead to the high-quality development of rural tourism, and their spatial distribution is influenced by a variety of factors. However, existing studies have neglected the fact that influencing factors can have different directions and effects in different geographic spaces. This study investigates 156 NKRTVs in the Yangtze River Delta region of China as the research object and employs ArcGIS spatial analysis technology to examine their spatial distribution characteristics. Additionally, two new indicators of land and culture are introduced to enhance the index system of influencing factors. A geographically weighted regression model is utilized to identify the spatial heterogeneity of various factors that affect the spatial distribution of NKRTVs. The results of this study indicate the following: (1) The spatial distribution of NKRTVs in the Yangtze River Delta region is characterized by "small clustering and large dispersion." The spatial distribution exhibits strong spatial correlation, with Shanghai serving as the primary spatial clustering core and Huangshan city forming a secondary spatial clustering subcore. The distribution of NKRTVs is relatively scattered in other areas, with obvious differences in the spatial distribution of cold and hot spots. (2) The results of the geographically weighted regression model show that with the change in spatial location, the influence effect of each influencing factor on the spatial distribution of NKRTVs has obvious spatial differences. Based on the spatial heterogeneity of the influencing factors, this study proposes targeted suggestions for the development of rural tourism in different regions.

## 1. Introduction

The development of the rural tourism industry has the potential to promote economic growth in rural areas, create employment opportunities, enhance farmers’ income, and improve their quality of life while also reducing urban‒rural disparities [1]. Since 2019, China has been identifying NKRTVs nationwide. As of 2022, three batches of national NKRTVs had been announced. NKRTVs have a relatively mature tourism industry with diversified business forms and well-established infrastructure. As such, they serve as benchmarks for driving high-quality rural tourism development [2]. The Yangtze River Delta region is an essential tourist destination in China, known for its abundant pastoral tourism resources and thriving tourism industry [3]. Investigating the spatial distribution pattern and the factors that influence NKRTV development in this region has significant practical value for promoting sustainable and high-quality rural tourism and achieving the strategic goal of rural revitalization.

Rural tourism research has been a key area of concern for academics at home and abroad, and foreign scholars believe that rural tourism and the traditional mass tourism industry can achieve good coexistence, provide differentiated tourism products, and generate different tourism attractions [4]. Studies have revealed that the spatial layout of rural tourism is closely linked with rural infrastructure improvements and tourism route planning. A rational spatial layout can help enhance the economic and social benefits of tourism [5]. Rural tourism destinations demonstrate characteristics of spatial autocorrelation in their spatial distribution, exhibiting territoriality and agglomeration [6]. The development of tourism in different regions is characterized by unevenness, with stark contrasts between coastal and inland rural areas [7] and economically developed and less developed regions [8].

Tourism geography research has focused on investigating the spatial relationships, distribution characteristics, and influencing factors of rural tourism destinations [9]. The utilization of GIS spatial analysis technology has been proven to be effective in identifying the spatial characteristics of tourist attractions, analyzing tourists’ spatial behavior and tourism intentions, and formulating competitive tourism development strategies [10]. For instance, by leveraging the spatial network structure of rural tourism, spatial analysis technology can identify rural tourism core villages and subcore villages, which can contribute to the rational allocation of rural tourism infrastructure and boost tourism efficiency [11]. Meanwhile, the development of rural tourism heavily relies on characteristic tourism resources, and previous studies have found that regions where traditional villages are clustered offer unique cultural resources for rural tourism development [12]. Moreover, renowned tourist attractions have significant impacts on the development of rural tourism. Tourist resources surrounding scenic spots and attractions are usually concentrated, with adequate infrastructure, and thus favor the development of rural tourism within a 5-kilometer radius of scenic spots [13].

Since the implementation of the policy of building NKRTVs in China, the spatial characteristics and influencing factors of such villages have been a research hot topic for domestic scholars. The research primarily focuses on a national scale, where different batches of NKRTVs are taken as research objects. The main approach is to study their spatial geographic characteristics and subsequently investigate the driving factors behind their formation. Studies have shown that the distribution of NKRTVs in China is uneven, with a higher concentration in the eastern region than in the western region [5] and remarkable clustering in major developed cities such as Beijing, Tianjin, and Shanghai [14]. The formation of the spatial pattern of NKRTVs can be divided into three stages: the initial stage involves a small number of villages with inherent geographical advantages conducive to the development of rural tourism; the developmental stage involves the government through policy encouragement and the improvement of infrastructure to promote the rise of rural tourism; the formative stage involves the nationwide selection and organization of NKRTVs, promoting the initial formation of the NKRTV pattern [15]. Furthermore, NKRTVs can be classified into diverse categories based on the specific tourism resources they depend on. For example, Ma Binbin et al. divided the country’s 320 NKRTVs into six major types: suburban recreation and leisure, scenic spot-based, cultural and folk tourism, ecological and leisure agriculture, specialty industry-led, and specialty resource development [8]. To examine the spatial distribution of rural tourism destinations, nuclear density analysis, the geographic concentration index, the nearest neighbor index, and geographic probes are commonly utilized as spatial analysis methods. It is worth noting that natural ecology, tourism resources, socioeconomics, and transportation are perceived as crucial determinants that influence the development of rural tourism [16–18].

However, previous studies have mainly employed a global analysis approach that overlays the influencing factors with the spatial distribution pattern at a macro level. This kind of analysis overlooks the local spatial correlation of variables, which can lead to different explanatory power of independent variables on dependent variables due to their location. This phenomenon is known as spatial nonstationarity [19]. In reality, variations in resource conditions and socioeconomic development across different regions result in complex and diverse influences on the spatial distribution of villages, which exhibit significant spatial heterogeneity characteristics. Geographically weighted regression (GWR) models utilize locally weighted regression analysis models, which consider the spatially varying relationships between dependent and independent variables based on the principle that these relationships change depending on spatial location. This approach offers a promising solution to address the complex and diverse influences on the spatial distribution of villages with significant spatial heterogeneity characteristics [20]. The GWR technique has been applied in various fields, including social and environmental sciences, such as village distribution [21], land use change [22], and urban house prices [23], as well as the effects of urban land use on air quality [24] and vegetation cover [25]. However, it has not been widely used in the study of the drivers of rural tourism development.

Therefore, this paper takes the NKRTVs in the Yangtze River Delta region as the research objects and utilizes the GWR model to analyze regional differences in the impact of various factors on the spatial distribution of NKRTVs. The study aims to provide insights into the underlying mechanisms behind the spatial distribution of NKRTVs in the region, with implications for decision-making guidance for the scientific development and layout optimization of rural tourism.

## 2. Materials and data

### 2.1 Study area

The Yangtze River Delta, situated in the eastern coastal region of China, comprises 41 cities, including Shanghai, and the provinces of Jiangsu, Zhejiang, and Anhui. It is one of the most economically developed regions in China, accounting for one-fourth of the country’s total economic volume as of 2019. The region boasts abundant tourism resources, a developed tourism transportation network, and a large tourism source market, which form a solid foundation for the development of rural tourism. The integrated development of urban and rural areas in the region is highly advanced in China, with relatively coordinated urban and rural development and a concentration of urban resources in rural areas to achieve a spillover effect. Rural tourism in the Yangtze River Delta started early and developed rapidly, becoming one of the most mature rural tourism destinations in China. As of 2022, there were 156 NKRTVs in the region, accounting for 13% of the total number, according to data from the Ministry of Culture and Tourism of China (https://mct.gov.cn/). With the onset of a new period of integrated development in the Yangtze River Delta, the region is striving to become a world-class rural tourism destination.

### 2.2 Data sources

The data utilized in this study are composed of two aspects. First, the dependent variable was derived from a list of 156 NKRTVs in the Yangtze River Delta region, sourced from the official website of the Ministry of Culture and Tourism of China (https://mct.gov.cn/) and covering the period up to 2021. The latitude and longitude coordinates of these locations were obtained using the Baidu Map Coordinate Picker website (https://api.map.baidu.com/lbsapi/getpoint/index.html), imported into ArcGIS 10.5 software, and projected onto the study area map. The kernel density value of the study unit was chosen as the dependent variable of the regression model in this paper, as it better reflects the sparsity of the spatial distribution compared to point data. Additionally, this approach facilitates subsequent regression analyses.

Regarding the independent variables, natural, demographic, economic, transportation, urban, and tourism resources are widely used in relevant studies. However, due to the emergence of new industries and business models in rural areas in recent years, the integration of agriculture, culture, and tourism in rural areas has achieved positive results [26]. Therefore, this study incorporates land and cultural factors into the analysis from the perspective of agricultural, cultural, and tourism integration. To establish a comprehensive index system for analyzing influencing factors, six systems were considered: topographic conditions, land resources, tourism resources, cultural resources, social economy, and transportation conditions (Table 1). To ensure the uniformity of each factor within a specific geographic area, the administrative boundary of the study area was employed as the base map. The fishnet creation tool in ArcGIS 10.5 was then utilized to divide the study area into 10 km x 10 km grids, consistent with existing research division standards [27, 28]. A total of 3,989 grids were generated as research analysis units.

**Table 1:** Spatial distribution of NKRTVs influences factor indicators and calculation methods.

	type	index	Calculation method	Data sources
Dependent variable	NKRTVs	Kernel density of NKRTVs	Grid Kernel Density Mean Value	Official website of the Ministry of Culture and Tourism of the People’s Republic of China (2022) (https://mct.gov.cn/)
Independent variables	topographic conditions	Elevation	Mean value of elevation in the grid	Geospatial data cloud website (2020) (https://www.webmap.cn/)
		Slope	Mean value of slope in the grid	
	Land resources	Arable land	The area of arable land within the grid	National Geographic Information Resources Directory Service System website (2020) (https://www.webmap.cn/)
		Woodland	Woodland area within the grid	
		Grassland	Grass area within the grid	
		Waters	Area of water within the grid	
	transport condition	Source market distance	The grid center is near and far from the nearest county-level administrative unit.	
		Accessibility	Road network density within the grid	
	Tourism resources	Number of A-class scenic spots	Grid Kernel Density Mean Value	Official website of the Ministry of Culture and Tourism of the People’s Republic of China (2020) (https://mct.gov.cn/)
	Cultural resources	Number of traditional villages	Grid Kernel Density Mean Value	Website of the Digital Museum of Traditional Villages (2020) (http://www.dmctv.cn/)
	Social economy	population	Population density within the grid	Resource and Environmental Science Data Registration and Publication System, 2019 Chinese Spatial Distribution Kilometer Grid Dataset (https://www.resdc.cn/)
		Gross Domestic Product (GDP)	GDP in the grid	Resource and Environmental Science Data Registration and Publication System: Spatial Distribution of China’s GDP in 2019 Kilometer Grid Data (https://www.resdc.cn/)
		GDP per capita	GDP per capita within the grid	2019 Annual Government Statistical Bulletin and Statistical Yearbook
		The primary sector as a share of GDP	The output value of the primary industry in the grid accounts for the proportion of GDP.	
		The tertiary sector accounts for the share of the GDP	The output value of the tertiary industry in the grid accounts for the proportion of GDP.	

Second, the grid-based analysis approach was utilized in ArcGIS 10.5 software, with a total of 3,989 grids used as the basic statistical unit. The kernel density data of NKRTVs and the data of influencing factors were linked to each grid using the spatial analysis tool. The grid spatial calculation method was applied to assign the data to each grid, ensuring data unification in one dataset [29]. Finally, a geographic element analysis database of NKRTVs in the Yangtze River Delta region was established to facilitate subsequent analysis. The calculation method and specific sources are shown in Table 1.

### 2.3. Research method

#### 2.3.1 Kernel density analysis

Kernel density analysis can identify the core of spatial aggregation. In terms of point set elements, the higher the probability of a particular probability event occurring in a point element is, the denser the point in space; in contrast, the lower the likelihood of an event, the more sparsely dense it is in the area [30]. This paper uses the kernel density analysis method to measure the spatial distribution of dispersion or clustering characteristics of NKRTVs. The formula is shown as follows:

$$f(x)=\frac{1}{nh}\sum_{i=1}^{n}k\left(\frac{x-x_{i}}{h}\right)$$ (1)

In the formula, *k* is the kernel function; *h* is the bandwidth; and the *x*−*x*_*i*_ is the distance from village *x* to village *x*_*i*_.

#### 2.3.2 Global spatial autocorrelation

Spatial autocorrelation analysis is based on element values and element locations to study the degree of spatial correlation between regions, and the global spatial autocorrelation metric coefficient Global Moran’s I is chosen in this study to analyze the spatial correlation of the distribution of NKRTVs in the study area; see Formula (2) [31]

$$I=\frac{n}{S_{0}}\frac{\sum_{i=1}^{n}\sum_{j=1}^{n}W_{i,j}(X_{i}-\bar{X})(X_{j}-\bar{X})}{\sum_{i=1}^{n}{\left(X_{i}-\bar{X}\right)}_{i}^{2}}$$ (2)

where *n* denotes the number of study units; *X*_*i*_ and *X*_*j*_ are the values of NKRTVs in regions *i* and *j*, respectively; *S*_0_ is the spatial weight matrix element sum; and *W*_*i*,*j*_ is the spatial weight matrix between elements *i* and *j*. The Moran’s I index takes the value interval of [–1,1]; if it is [0,1], then it indicates a positive correlation, elements in If [0,1] indicate a positive correlation, and the details tend to be aggregated in space; if [–1,0] shows a negative correlation and the details tend to be dispersed in the area, a value equal to 0 indicates that there is no spatial correlation and the distribution is random.

#### 2.3.3 Getis-Ord Gi* index

The Getis-Ord Gi* index can precisely identify statistically significant high-value and low-value clustering areas, i.e., hot and cold areas of spatial distribution [32]. In this study, the Getis-Ord Gi* index was used to identify the hot and cold zones of the spatial distribution of NKRTVs. The spatial distribution pattern of NKRTVs was analyzed in Eq (3).

$$G_{i}^{*}=\frac{\sum_{j=1}^{n}W_{i,j}X_{j}-\bar{X}\sum_{j=1}^{n}W_{i,j}}{\sqrt{\frac{\sum_{j=1}^{n}X_{j}^{2}}{n}-{\left(\bar{X}\right)}^{2}}\sqrt{\frac{n\sum_{j=1}^{n}W_{ij}^{2}-{\left(\sum_{j=1}^{n}W_{ij}\right)}^{2}}{n-1}}}$$ (3)

Where *X*_*j*_ is the location of the *j*th study unit, *n* is the number of study units, and *W*_*i*,*j*_ is the spatial weight matrix; in the statistically significant case, high $G_{i}^{*}$ values represent high-value clustering areas, and low $G_{i}^{*}$ values represent low-value clustering areas.

#### 2.3.4 Geographically weighted regression (GWR)

The GWR model is an improvement of ordinary least squares (OLS) linear regression, which mainly considers the spatial weight of geographic coordinates, i.e., the regression coefficients will have different directions of action and influence effects with location changes to explain the spatial heterogeneity of influencing factors [33]. The model expression is:

$$y_{i}=\beta_{0}(\mu_{i},\vartheta_{i})+\sum_{k=1}^{n}\beta_{k}(\mu_{i},\vartheta_{i})x_{ik}+\varepsilon_{i}$$ (4)

In the formula, *y*_*i*_ is the global variable; *x*_*i*_ is the value of the *k*th independent variable; (*μ*_*i*_, *ϑ*_*i*_) is the geographical coordinate of I; *β*_0_(*μ*_*i*_, *ϑ*_*i*_) is the estimate of the constant term; *n* is the number of independent variables; *β*_*k*_(*μ*_*i*_, *ϑ*_*i*_) is the estimate of the coefficient of the *k*th variable; and *ε*_*i*_ is the error term.

## 3. Spatial distribution characteristics of NKRTVs

**Fig 1:**
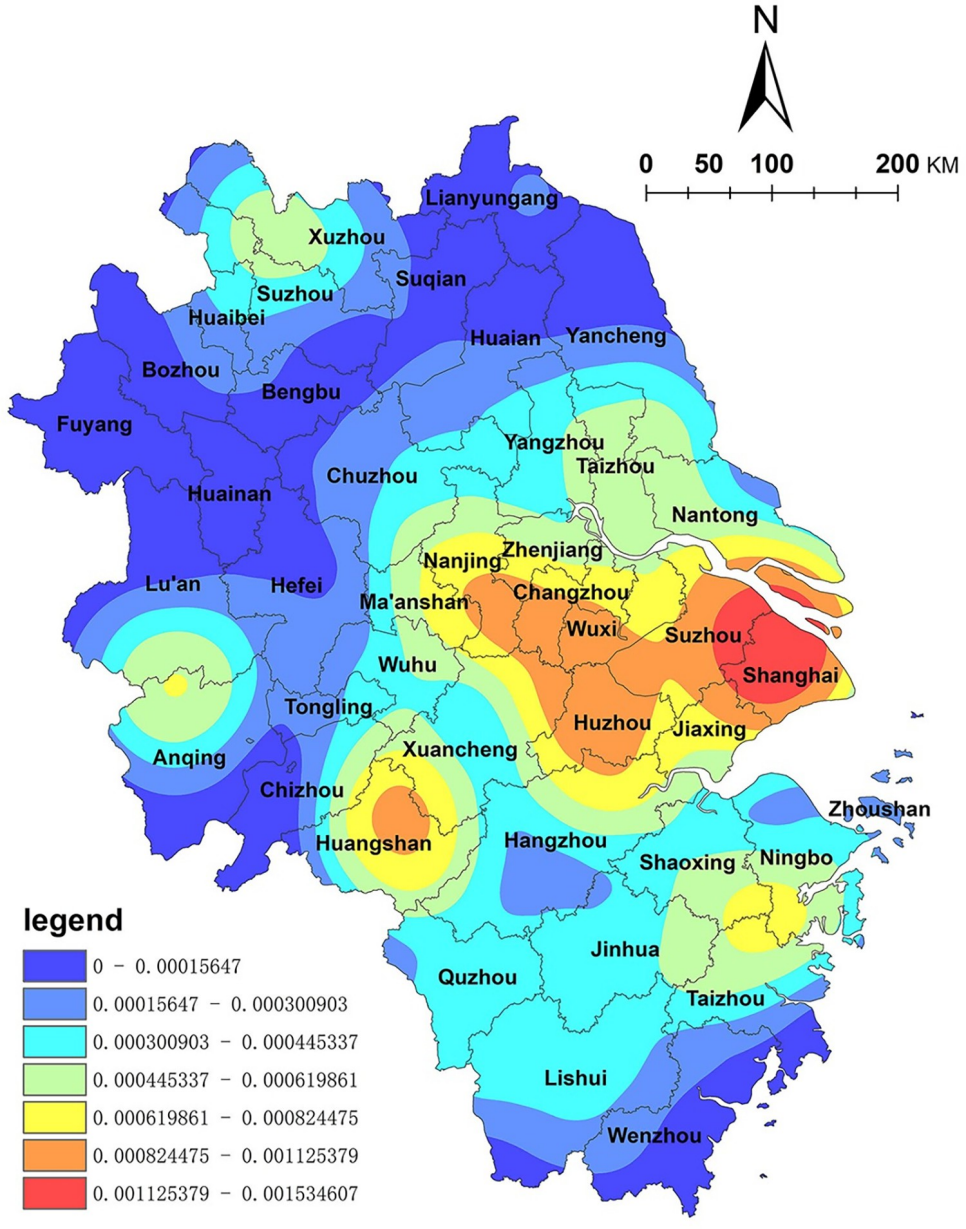
Spatial distribution density map of NKRTVs. (The authors used ESRI’s ArcGIS 10.5 software to draw).

Using the administrative boundaries of the Yangtze River Delta region as a masking range, a kernel density analysis was performed on the NKRTVs within the study area, and a spatial distribution density map of NKRTVs was obtained (Fig 1). The results indicate that the spatial distribution of NKRTVs in the Yangtze River Delta region is generally characterized by "small clustering and large dispersion." The high-density area of the spatial distribution of NKRTVs is mainly centered on Shanghai and spreads to the Taihu Lake Plain areas, such as Suzhou, Huzhou and Jiaxing. The Yangtze River Delta region is one of the most economically active, open and innovative regions in China, but the problem of unbalanced development between provinces and between urban and rural areas is also prominent. China has been implementing a rural revitalization strategy since 2018 to provide material security for achieving common prosperity for everyone. Shanghai and Suzhou, as the most socio-economically developed regions in the Yangtze River Delta, have strong economic power. In order to implement the strategy of rural revitalization and achieve common prosperity for everyone, the countryside, as a scarce resource in megacities, has received very large economic investments, especially in tapping rural resources to develop rural tourism. In this context, the multiple functions of the countryside and the ecological, leisure and cultural values of the countryside have been fully developed, and a number of rural tourism destinations with diversified types and distinctive features have been built. Moreover, this area has been very affluent since ancient times, and the area south of the lower reaches of the Yangtze River has many ancient water towns, such as Zhouzhuang Ancient Town and Wuzhen. Simultaneously, a considerable number of rural bed and breakfasts (B&Bs) have clustered around the Moganshan International Tourism Resort, which is a novel way of using vernacular architecture in the process of rural tourism transformation and upgrading. It is an emerging tourism format, and the area around Moganshan is one of the most famous homestay clusters in China. In the Huangshan city area of Anhui Province, a secondary spatial agglomeration core has emerged, which is an area enriched with high-quality tourism resources, such as the unique natural scenery of the Huangshan Scenic Area and ancient villages such as Hongcun and Xidi [34].

**Fig 2:**
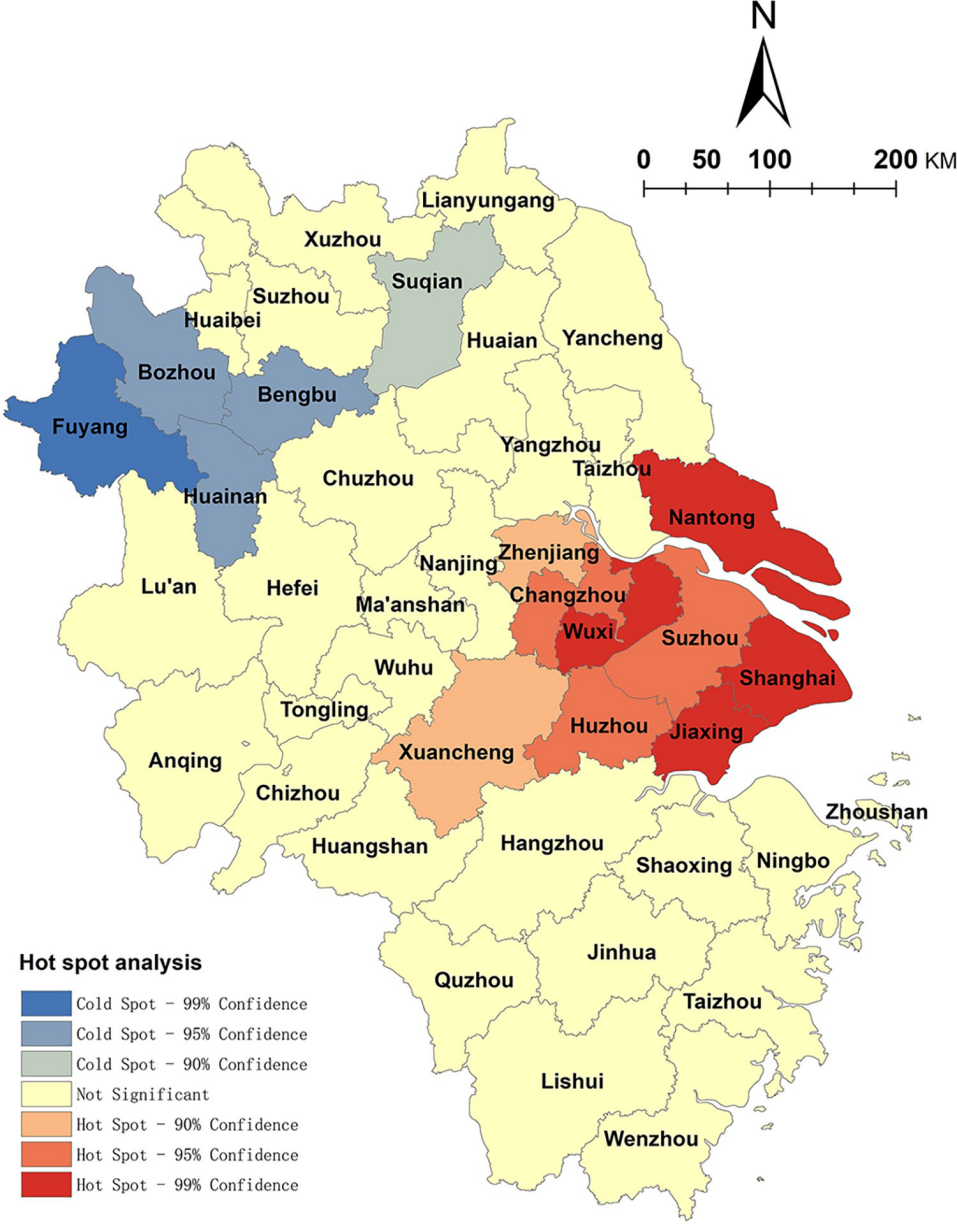
Spatial distribution map of cold and hot spots in NKRTVs. (The authors used ESRI’s ArcGIS 10.5 software to draw).

This paper uses ArcGIS10.5 software to calculate the global Moran index estimate of the spatial distribution of RTKVs, which is 0.809. The average distribution statistic Z value is 7.546, with a P value less than 0.01, which passes the 99% significance level test. This means that the spatial distribution of NKRTVs in the Yangtze River Delta region has a strong spatial autocorrelation, i.e., the spatial distribution of NKRTVs. The parts with relative density values are also closer in space. There is a greater possibility that they are interrelated, and the future should focus on the uneven development of rural tourism in the region. The results of the Getis-Ord Gi* index analysis (Fig 2) show that NKRTV hot spots are mainly distributed in Shanghai, Suzhou, Wuxi, and other cities, and the cold spots are distributed in Fuyang and Bozhou in Anhui Province, which is consistent with the results of the nuclear density analysis. There is autocorrelation in the spatial distribution of NKRTVs, and the effects of spatial autocorrelation analysis also provide a basis for this research to study the influencing factors of the distribution of NKRTVs using the geographically weighted regression analysis model.

## 4. Spatial heterogeneity analysis of factors influencing spatial distribution

### 4.1 Comparison of GWR model and OLS model regression results

Before operating the GWR model, the OLS regression model should be used to test the influence and degree of fit of each independent variable on the overall spatial distribution of NKRTVs. In this paper, the factor base data of the respective variables in Table 1 were standardized to eliminate the dimensional differences of the data, imported into ArcGIS10.5 software, and calculated using the OLS regression analysis model in the spatial statistics tool. The calculated results showed that the R^2^ and adjusted R^2^ of the model were 0.468 and 0.465, respectively, and the AICc value was -55419.24.

To prevent multicollinearity and ensure the accuracy of the analysis results, it is necessary to exclude the factors with VIF values greater than 3.5 and the influencing factors with insignificant coefficients. Finally, eight indicators were determined as influencing factors for geographic weighted regression analysis: the area of arable land within the grid, the size of water within the grid, the average value of nuclear density of A-class scenic spots in the grid, the average atomic density of traditional villages in the grid, GDP per capita within the grid, the output value of the primary industry in the grid accounting for the proportion of GDP, the output value of the tertiary sector in the grid accounting for the balance of GDP, and distance from the grid center to the nearest city at or above the county level (Table 2).

**Table 2:** OLS model analysis results.

variable	coefficient	VIF
Mean value of elevation in the grid	0.77	3.32
Mean value of slope in the grid	0.00*	5.94
The area of arable land within the grid	0.00*	2.70
Woodland area within the grid	0.00*	8.14
Grass area within the grid	0.91	1.12
The area of water within the grid	0.00*	1.28
The average value of nuclear density of A-class scenic spots in the grid	0.00*	2.50
the average atomic density of traditional villages in the grid	0.00*	2.40
Population density within the grid	0.97	9.29
GDP in the grid	0.33	2.16
GDP per capita within the grid	0.00*	3.20
The output value of the primary industry in the grid accounts for the proportion of GDP	0.00*	2.43
The output value of the tertiary industry in the grid accounts for the proportion of GDP	0.00*	1.24
Distance from the grid center to the nearest city at or above the county level	0.01*	1.46
The density of the road network within the grid	0.09	1.04

* Note. An asterisk (*) indicates that the coefficient is statistically significant, i.e., p<0.05.

This paper uses the GWR model to simulate the differences in the effects of different variables on the spatial distribution of NKRTVs in other regions and chooses the AICc method to determine the optimal bandwidth for GWR regression analysis in the ArcGIS10.5 platform. The calculated results show that the R^2^ and adjusted R^2^ of the GWR model are 0.672 and 0.668, respectively, and the AICc value decreases to -57273.06. Compared with the OLS model, the R^2^ and adjusted R^2^ of GWR are more significant than the operation results of the OLS model, and the value of AICc decreases by more than 3, indicating that the GWR model has better fitting results (Table 3).

**Table 3:** Comparison of OLS model and GWR model diagnostic indicators.

Diagnostic metrics	OLS model results	GWR model results
R^2^	0.468	0.465
Adjusted R^2^	0.672	0.668
AICc	-55419.24	-57273.06

### 4.2 Spatial heterogeneity analysis of influencing factors

**Fig 3:**
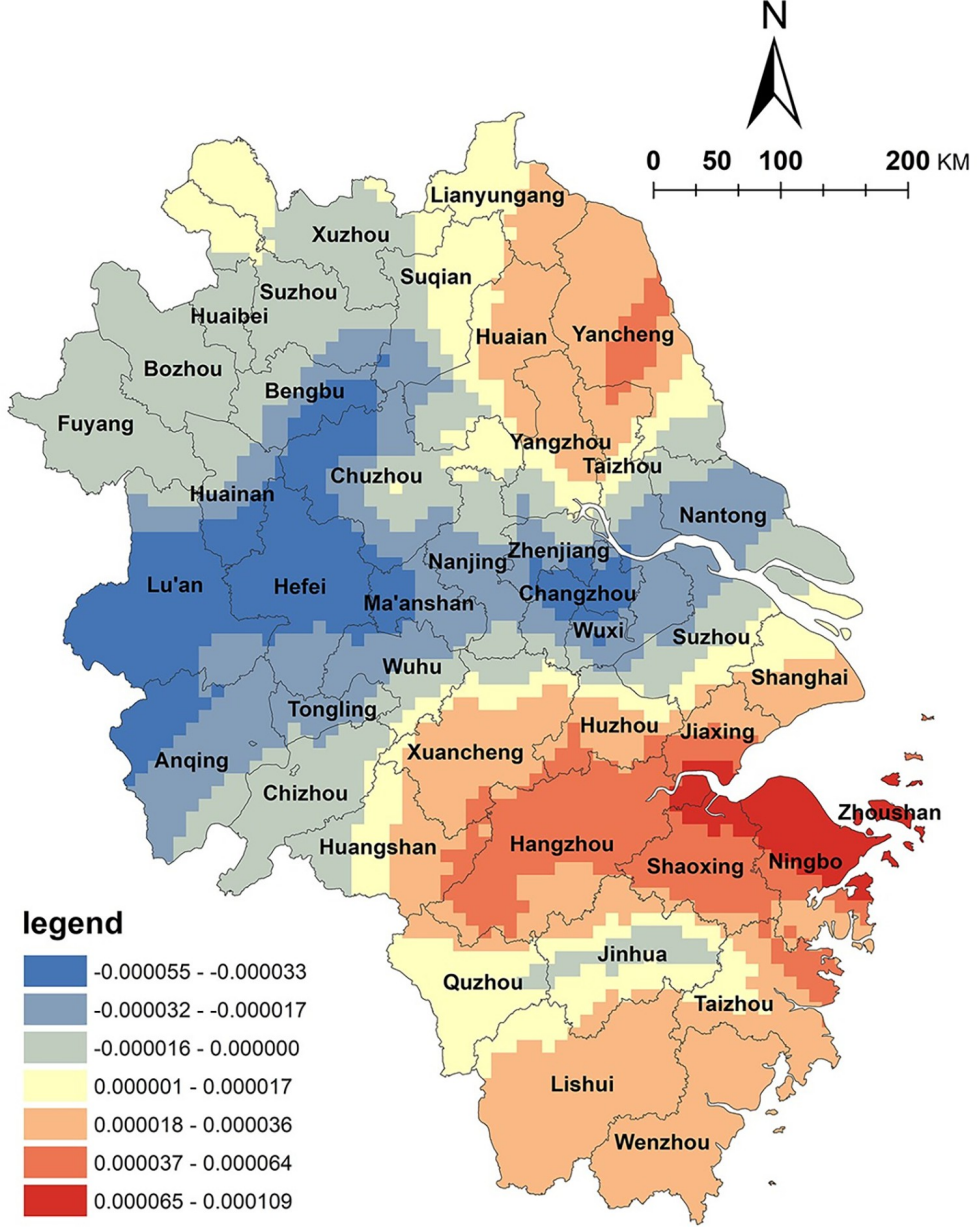
Analysis of arable land factors. (The authors used ESRI’s ArcGIS 10.5 software to draw).

The results of the GWR model indicate a positive correlation between arable land resources and the spatial distribution of NKRTVs in Zhejiang Province, with high-value areas mainly located in the eastern regions, such as Ningbo and Zhoushan (Fig 3). However, in the northern plain and Taihu plain areas of Anhui Province, a negative correlation was observed. Zhejiang Province has a typical mountainous terrain with complex topography. The area of arable land only accounts for 10% of the total land area and is dispersed in valleys and basins. The diverse topography has created rich agricultural natural landscapes and various agricultural production types in the region. In recent years, Zhejiang Province has actively promoted the integration of agriculture and tourism, accelerated rural land circulation, improved rural infrastructure, and vigorously developed rural tourism. Under the mechanism of multiple stakeholders, the utilization of cultivated land has become more diversified. For example, in rural tourism development, modern agricultural production, agricultural product processing, and agricultural sightseeing experiences are conducted on cultivated land, highlighting agricultural tourism and leisure functions and providing basic support for the development of tourism products such as vacationing, health preservation, sports, leisure agriculture, and cultural experiences. Therefore, the level of agricultural and tourism integration in this region is relatively high, and there is a positive correlation between arable land resources and the spatial distribution of NKRTVs. In contrast, the northern plain and Taihu plain areas of Anhui Province are vast plains with abundant land resources, which are very suitable for grain cultivation and belong to the national grain-producing regions. However, the arable land in this area is mainly used for grain production, and the importance of grain output has received more attention. Farmers generally need more awareness of transformation and development. The overall level of integration of agriculture and tourism could be higher.

**Fig 4:**
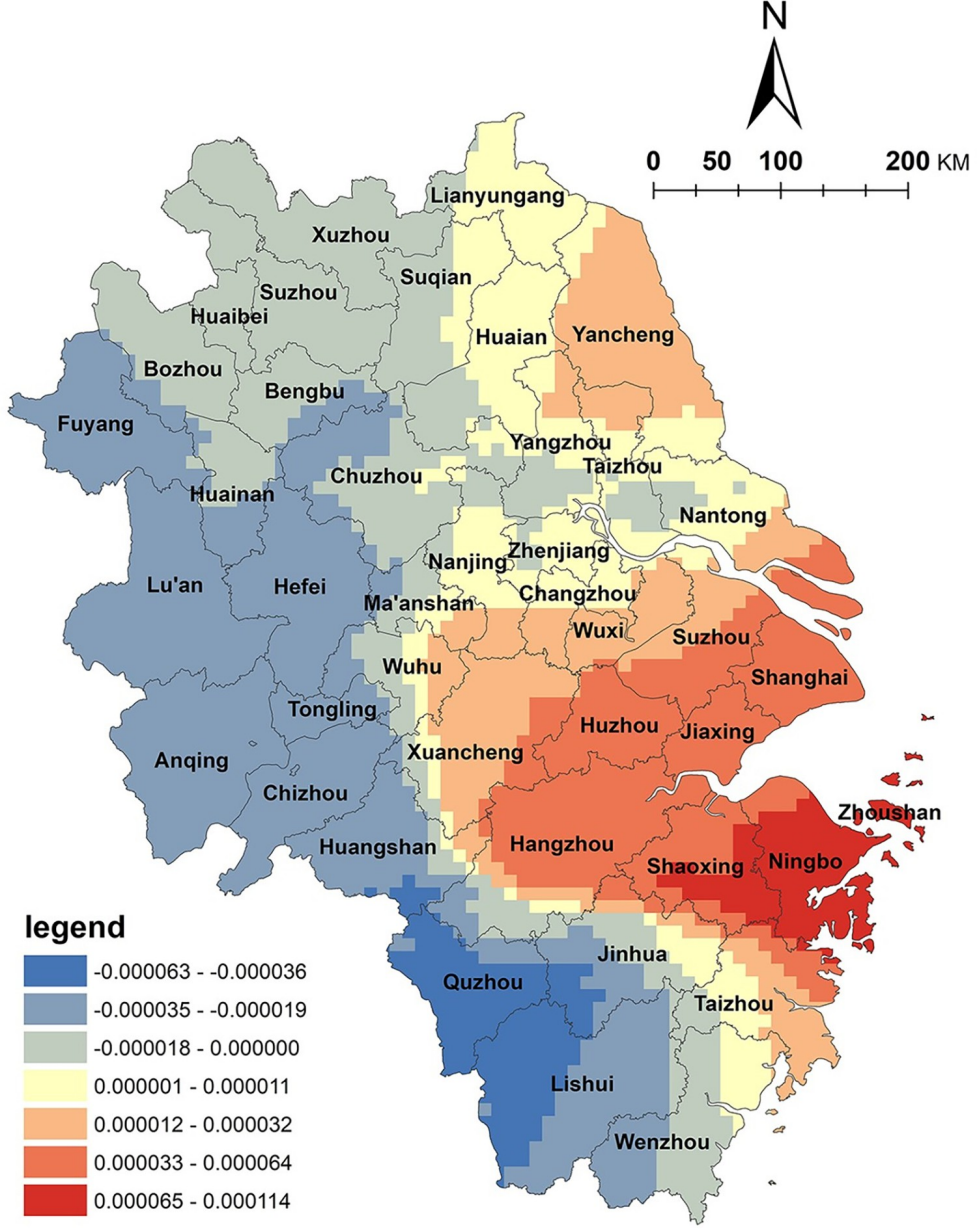
Analysis of water factors. (The authors used ESRI’s ArcGIS 10.5 software to draw).

Water factors play a vital role in creating a conducive rural tourism environment. Analysis of the water factor (Fig 4) reveals a significant east‒west regional disparity in the impact of water area size on the spatial distribution of NKRTVs, with a positive correlation in the eastern portion of the Yangtze River Delta region and a negative correlation in the western part of Anhui Province and the southwestern region of Zhejiang Province. The eastern region of the Yangtze River Delta, such as Hangzhou Bay and Taihu Plain in Zhejiang Province, boasts a dense water network and favorable hydrological conditions. The relationship between rural tourism and the water landscape is closely intertwined, and rural tourism destinations in this region rely on the abundant water network to form numerous ancient towns, which are typical of the southern region of China. In contrast, western Anhui Province and southwestern Zhejiang Province, which exhibit negative correlations, are mountainous and hilly areas with limited water areas and sparse water networks, which negatively affect the spatial distribution of NKRTVs.

**Fig 5:**
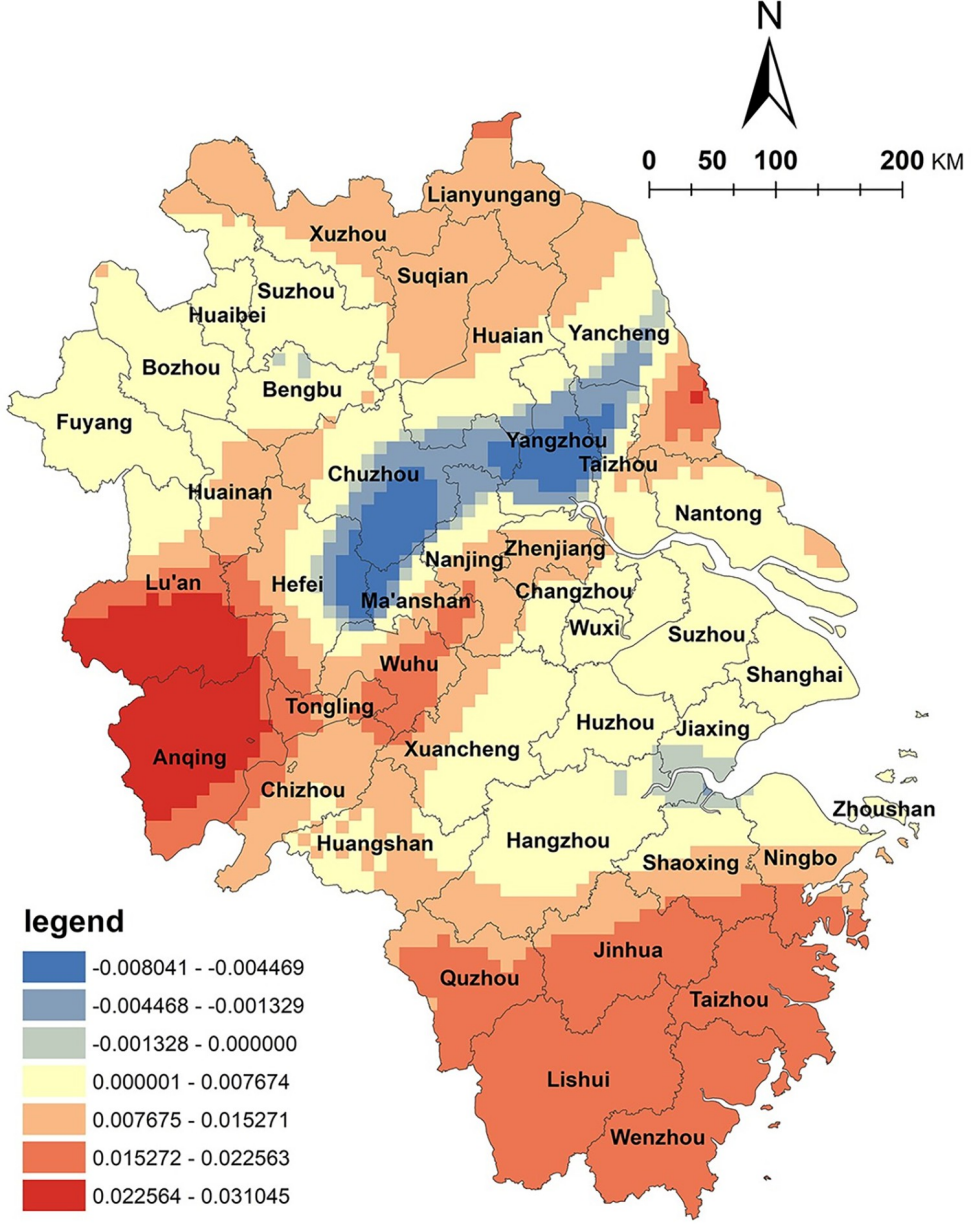
Analysis of factors of A-class scenic spots. (The authors used ESRI’s ArcGIS 10.5 software to draw).

This study introduces the spatial distribution density of A-level tourist attractions to reflect the regional tourism resource endowment. As shown in Fig 5, the spatial distribution of tourism resources in the Yangtze River Delta region generally shows a positive correlation with the spatial distribution of NKRTVs, with only negative correlations in cities such as Chuzhou and Yangzhou. Overall, rural tourism development is highly dependent on local tourism resources, which is consistent with reality. The complementarity between NKRTVs and tourist attractions enables them to share customers and achieve close cooperation [35]. The positively correlated high value areas appear in the cities of Lu’an and Anqing in Anhui, which belong to the core area of the Dabie Mountain revolutionary base area and are rich in red-themed tourism resources. Rural tourism development relies mainly on the red cultural tourism resources here. The negative correlation area is located in the cities of Chuzhou and Yangzhou, where tourism resources are abundant but mostly located in urban areas, and rural tourism resources are relatively scarce and not distinctive. There is little interaction between NKRTVs and A-level tourist attractions in this area.

**Fig 6:**
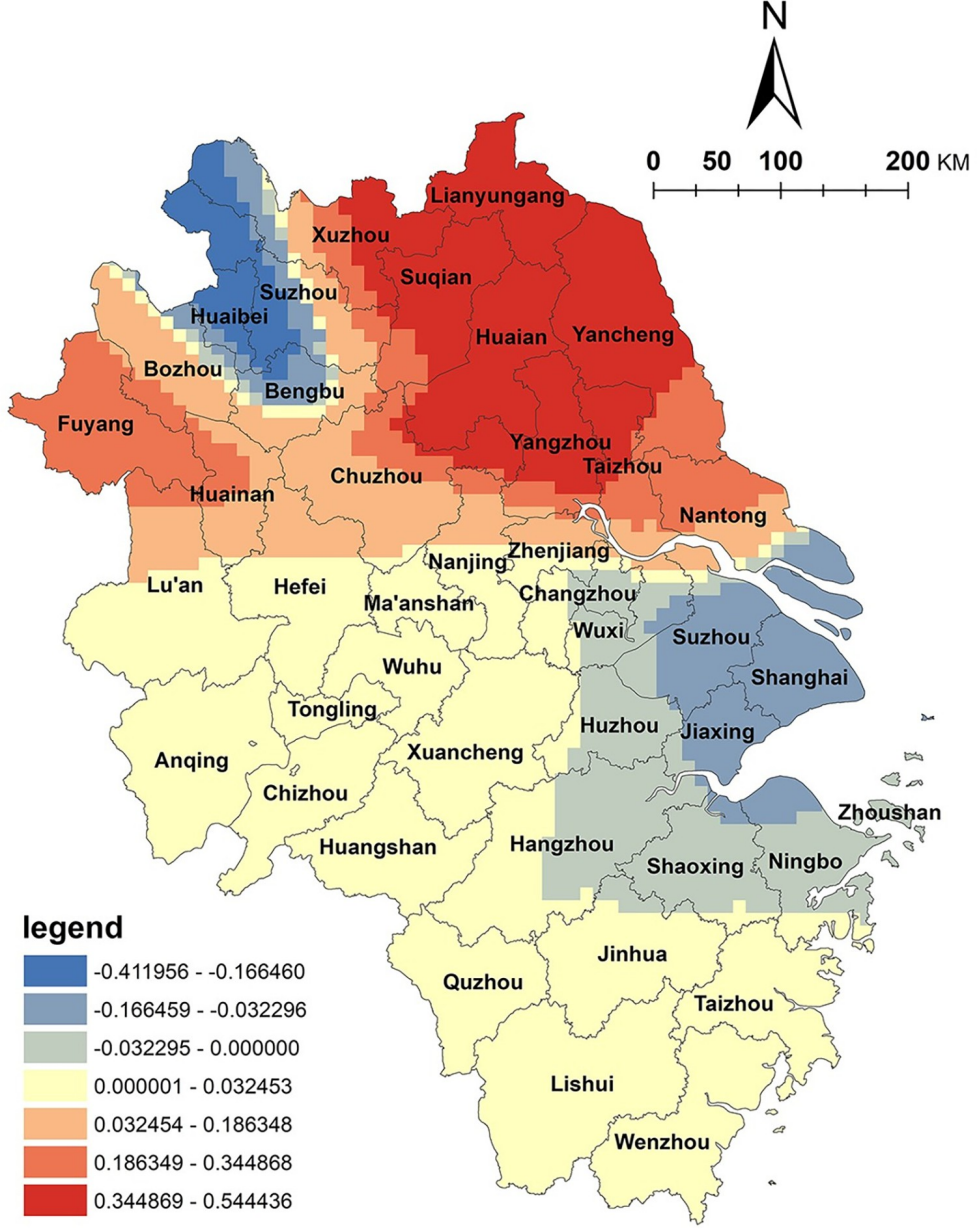
Analysis of traditional village factors. (The authors used ESRI’s ArcGIS 10.5 software to draw).

This study utilizes the spatial distribution density of traditional villages to reflect the rural cultural environment in the Yangtze River Delta region. The results are shown in Fig 6. The distribution density of traditional villages and the spatial distribution of NKRTVs show a significant positive correlation in Jiangsu, Zhejiang and Anhui provinces. The areas with high positive correlation values are in northern Jiangsu Province and northern Anhui Province. These findings suggest that the cultural resource factors found here have a positive influence on rural tourism development. In the context of cultural tourism integration, cultural resources are vital in supporting rural tourism development, and developing rural tourism based on traditional villages is a specific measure to revitalize the rural economy [36]. Traditional villages, an important cultural heritage left by China’s agricultural civilization, are the most important cultural tourism resources in rural areas, serving as important carriers for rural tourism development due to their rich historical and cultural connotations and cultural landscapes. For example, Gushan, Dongluo, and Liuwei villages in northern Jiangsu Province are NKRTVs and are recognized as traditional villages in Jiangsu Province. Rural tourism and traditional village resources have established good interaction. In fact, many rural areas rely on local history, culture, and traditional folklore to develop rural tourism, which enhances their cultural attractiveness and competitiveness. Two typical development models based on traditional villages in the mountainous region of southwest Zhejiang Province and red tourism resources in the Dabie Mountain region of Anhui Province can serve as examples.

**Fig 7:**
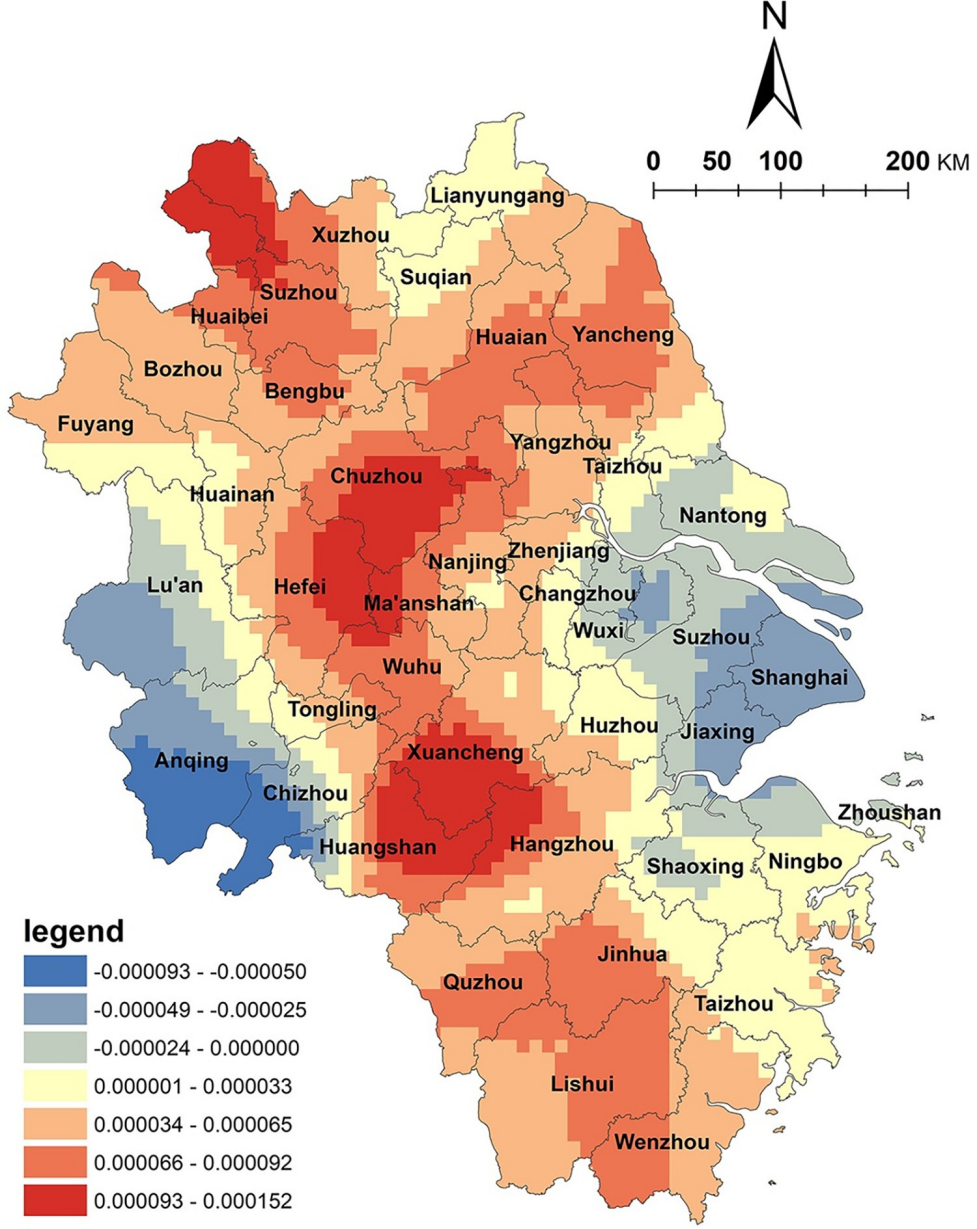
Analysis of GDP per capita factor. (The authors used ESRI’s ArcGIS 10.5 software to draw).

The level of economic development is a crucial basis for the development of NKRTVs. Previous studies have found that the distribution density of NKRTVs is positively correlated with the level of economic development; thus, the distribution density of NKRTVs in regions with higher levels of economic development is also high [9]. However, based on spatial heterogeneity, it was found that the spatial distribution of GDP per capita and NKRTVs in the Yangtze River Delta region was negatively correlated in the large cities of Shanghai, Suzhou and Nanjing, which are economically developed, highly modernized and populated, while the rural areas around these large cities showed a positive correlation. A band of positively correlated high value distribution is formed around major cities such as Shanghai, Suzhou, Nanjing and Hangzhou (Fig 7). It can be understood that on a regional scale, a high level of GDP per capita does not mean that the spatial distribution density of NKRTVs will be equally high; rural tourism is based on economically developed large cities as source markets around which a rural tourism belt is formed. It can be seen that at the national scale, the level of economic development determines the number and scale of NKRTVs, but when the research scale is reduced, it is found that an appropriate distance is still maintained between rural tourism destinations and cities, and this relationship cannot be simply applied.

**Fig 8:**
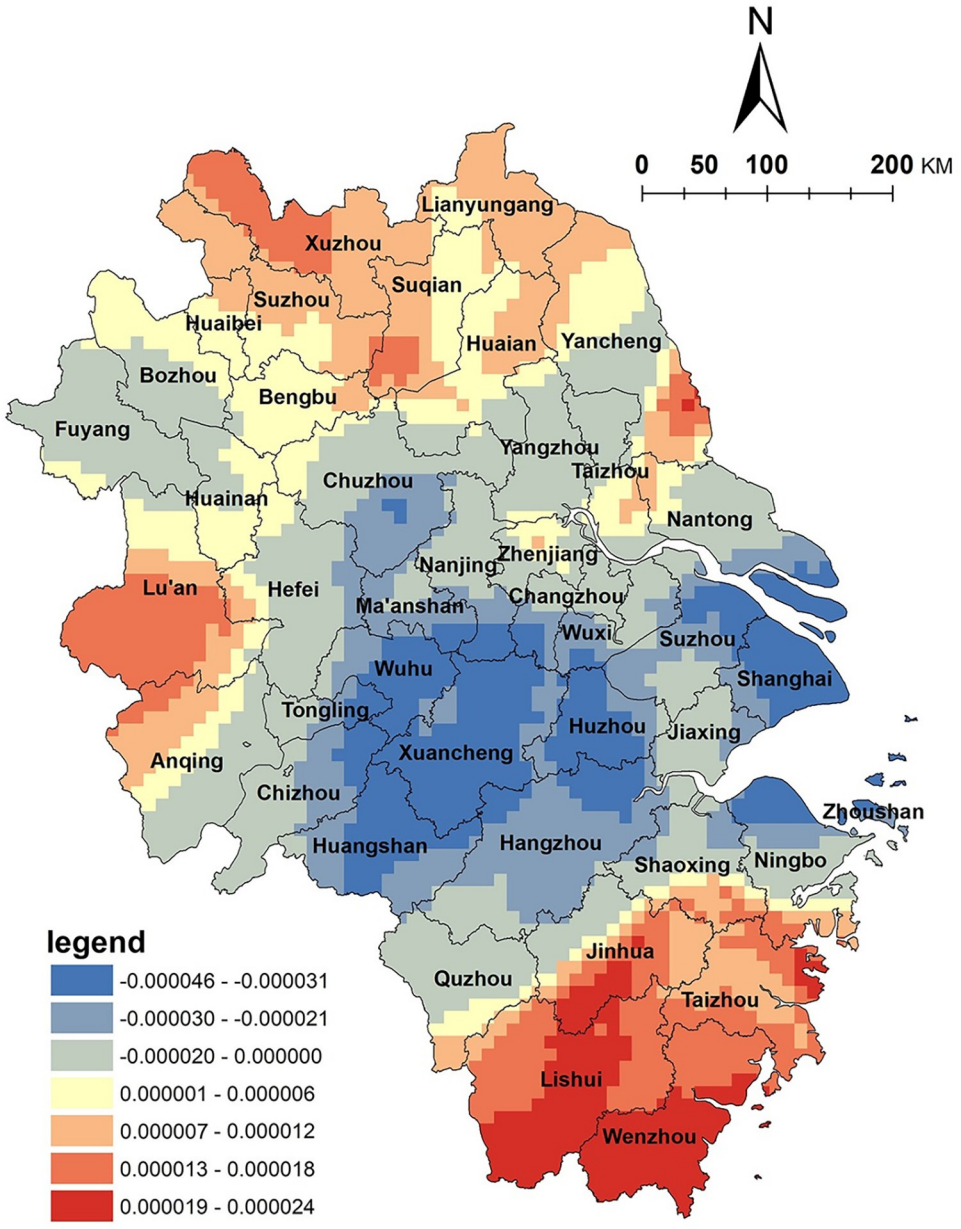
Analysis of source market factors. (The authors used ESRI’s ArcGIS 10.5 software to draw).

The transportation condition factor is mainly the distance between the source market and the NKRTVs. Urban residents are the main customer group for rural tourism, and the distance between tourist villages and urban areas is an important location factor affecting the development of rural tourism [8]. In this paper, the distance to the nearest county-level administrative unit is used to represent customer market factors. As shown in Fig 8, in large city areas such as Shanghai, Suzhou, Hangzhou, Wuxi and Nanjing, the proximity to the nearest county-level administrative unit has a negative correlation with the spatial distribution of NKRTVs, which can be interpreted as the farther the distance from the developed large cities, the lower the density of the distribution of NKRTVs. The densely populated and economically developed cities in the study area have a strong demand for rural leisure tourism, mostly as the main source of rural tourism, around which a number of peri-urban rural tourism destinations have been formed, which is also consistent with existing research [37]. On the other hand, a positive correlation was observed in economically underdeveloped regions such as southwest Zhejiang, north Anhui, and north Jiangsu, which are mainly mountainous and hilly areas and plains. It can be understood that in economically underdeveloped areas, the farther away from the city, the better preserved the ancient villages and vernacular cultures are. These are special tourism attractions, and they rely on these cultural and agro-tourism resources. A number of key villages of characteristic resource-developed rural tourism have been developed, such as the traditional village agglomeration in Lishui City, southwestern Zhejiang Province.

**Fig 9:**
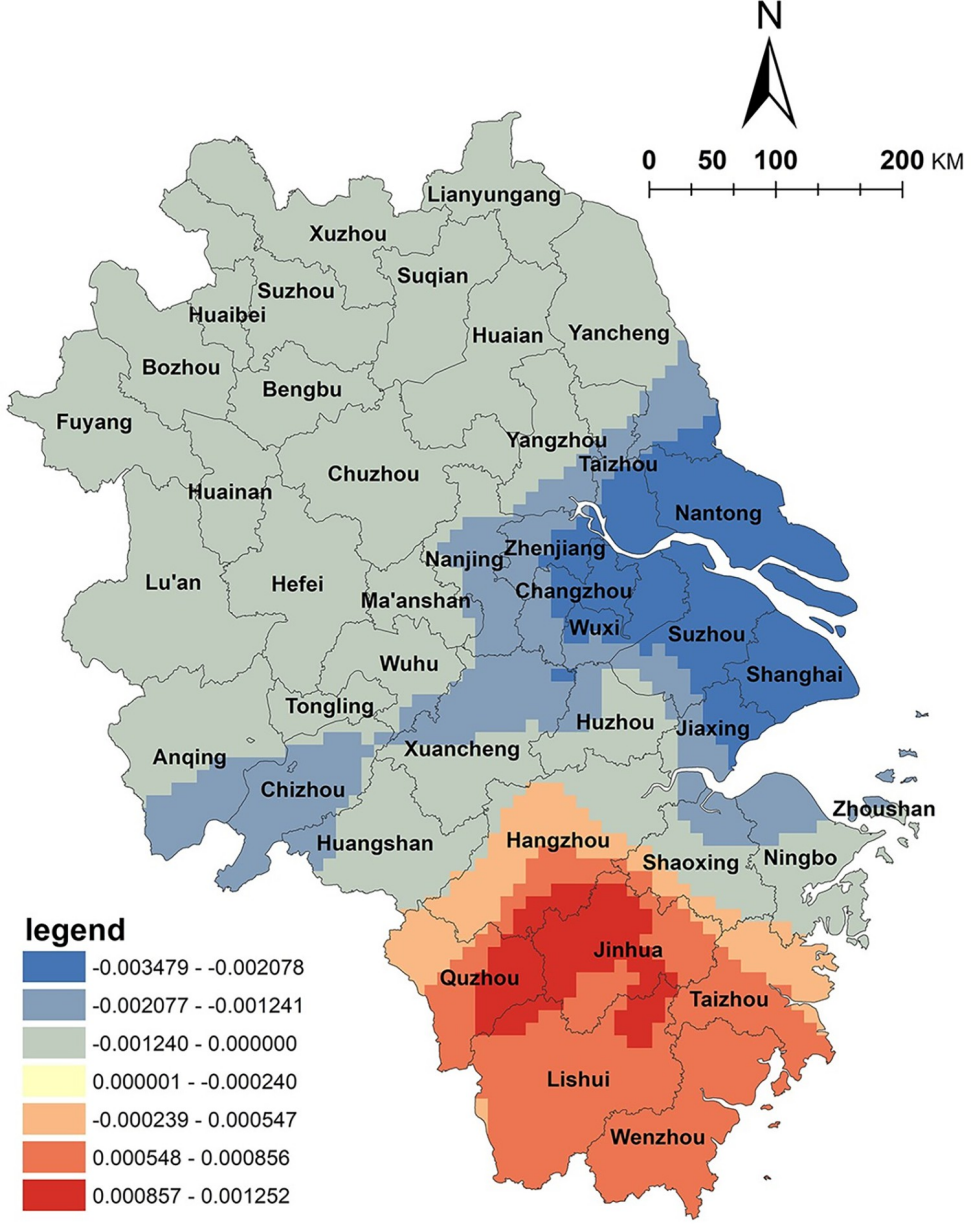
Analysis of primary industry factors. (The authors used ESRI’s ArcGIS 10.5 software to draw).

The revitalization of industries is the material foundation for achieving rural revitalization, and the development of rural tourism must rely on the advantages of characteristic rural industries. It was found that the proportion of primary industry output value to GDP and the spatial distribution density of NKRTVs had a positive correlation in the southern part of Zhejiang Province (Fig 9) and a negative correlation in the peripheral area of Shanghai and the plain area of the northern part of Anhui Province. The southern part of Zhejiang Province has a mountainous terrain, and various types of agriculture, forestry, animal husbandry, and fishing are distributed crossly, and the combination is relatively good. At the same time, Zhejiang Province actively promotes the construction of rural leisure agriculture, and the level of agricultural modernization is relatively high, providing a resource foundation for the development of rural tourism. Therefore, the increase in the proportion of the value added by the primary industry to GDP has a significant positive impact on the development of rural tourism in this region. In contrast, the negatively correlated regions are divided into two situations: the economically developed surrounding areas of Shanghai and the northern plain areas of Anhui Province, where agricultural development is the main focus. In the surrounding areas of Shanghai, the proportion of the value added by the primary industry to GDP is low, but there are many NKRTVs, mainly because of the economic development of the region. Although the added value of the primary industry is low, the strong demand of the passenger market in big cities has led to a large investment in rural tourism, and many types of rural tourism products can be developed, such as ancient towns and Moganshan countryside lodgings. Unlike around Shanghai, the primary industry in the plains of northern Anhui accounts for a high proportion of GDP, while the number of NKRTVs is small. This is mainly because of the single type of rural industry in the region. Traditional food crop cultivation is the characteristic of economic development, but food cultivation is at the level of the production function and economic function of agriculture, and the cultural function, tourism function and leisure function of the primary industry have not been effectively developed. Therefore, the output value of the primary industry accounts for a high proportion of the GDP, while the rural tourism service industry has not been developed, which is the dilemma faced by the transformation and upgrading of agriculture in the region.

**Fig 10:**
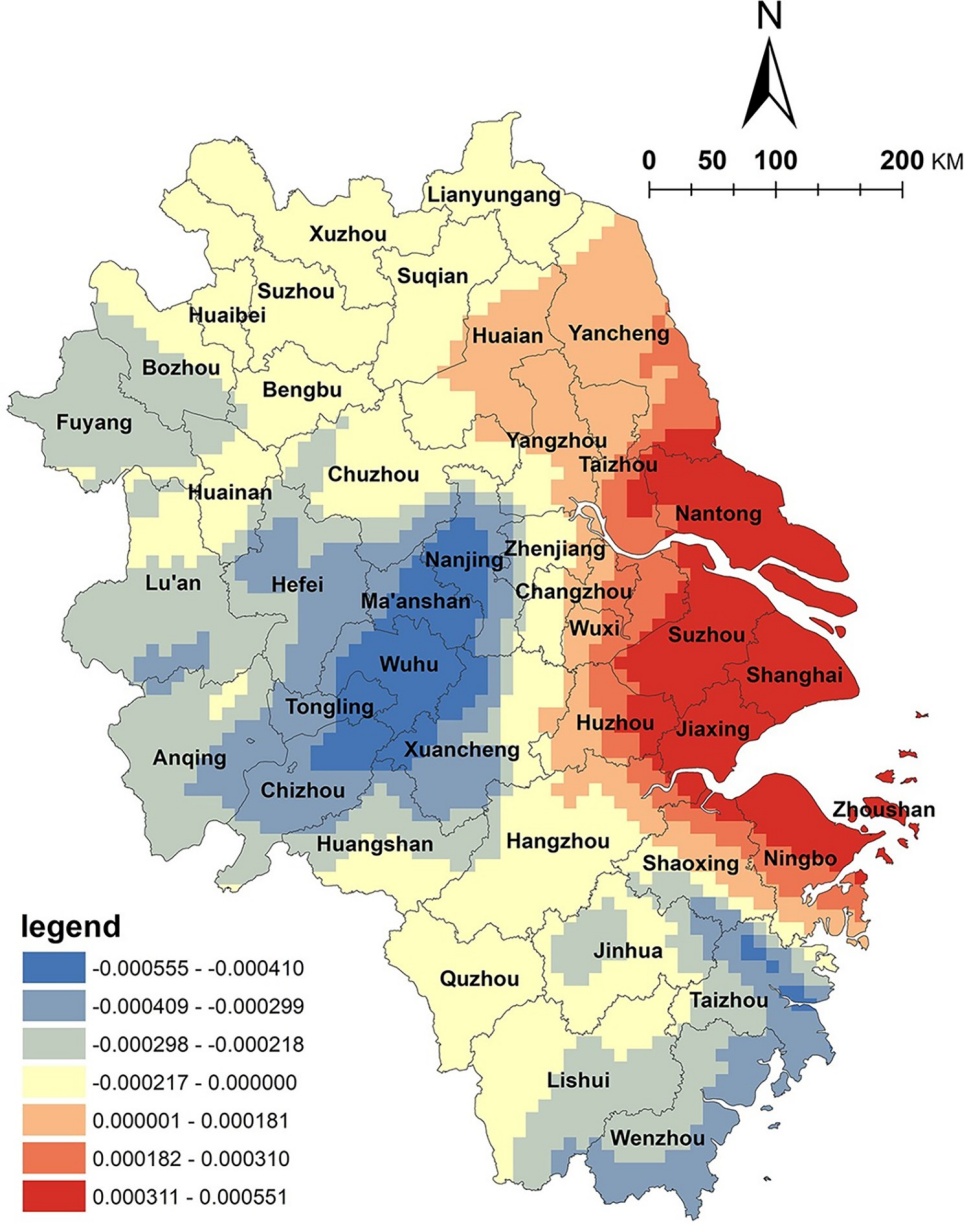
Analysis of tertiary industry factors. (The authors used ESRI’s ArcGIS 10.5 software to draw).

In addition, the regions where the proportion of the value added by the tertiary industry to GDP is positively correlated with the spatial distribution of NKRTVs are mainly located in Shanghai and its surrounding areas (Fig 10). In this region, the density of NKRTVs is high, the service industry is developed, and there is a positive and active impact between the two.

## 5. Discussion

As of August 2022, China has announced three batches of 1,199 NKRTVs, with the number continuing to increase. The spatial distribution of NKRTVs is affected by various complex and diverse factors, including regional resource endowments and socioeconomic development conditions. The impact of each influencing factor may vary with geographical location, and the spatial nonsmoothness of these factors has often been overlooked in previous studies. This paper’s findings suggest that the spatial performance of arable land and water area, A-class scenic spots, traditional villages, GDP per capita, distance to source markets, and the proportion of primary and tertiary industries in GDP on the spatial distribution of NKRTVs varies significantly. These differences are particularly noticeable between economically developed and less developed areas, as well as between plain areas and mountainous or hilly areas. For example, the arable land factor can have both positive and negative impacts in the plains of northern Anhui and the mountains of southwestern Zhejiang, resulting in distinct patterns of rural tourism development in these two regions. This study provides a valuable reference for promoting sustainable rural tourism development in different areas. Based on the spatial heterogeneity of the factors influencing the distribution of NKRTVs, this paper proposes the following recommendations.

1. The development of rural tourism requires clear classification guidance and accurate planning. To achieve this, it is essential to identify the types of rural tourism villages and the factors affecting the development of rural tourism in different regions. Adherence to the principle of "adapting measures to local conditions, distinctive characteristics, and integrating agriculture, culture, forestry, tourism, and health" is crucial. Additionally, targeted development strategies should be formulated for different villages in mountainous or plain areas. Full utilization of local resources is also necessary to develop local characteristic rural tourism products and promote sustainable development. Ultimately, the aim is to achieve balanced rural tourism development within the region.

2. To fully exploit the multifunctionality of agriculture, it is essential to cultivate innovative agricultural concepts. In addition to providing agricultural and ecological products, agriculture possesses numerous other functions, such as education, culture, landscape, leisure, and health care. Despite the vast arable land in the northern plain area of Anhui, the full potential of agriculture’s multifunctionality remains underutilized. To address this, rural tourism in the area can be closely integrated with the planting and breeding industry and farming culture. This integration can leverage the agricultural landscape and cultural creativity to enhance agrarian production and cultivate characteristic family farms, pastoral complexes, shared farms, and other rural tourism products. By maximizing land resources and activating rural production factors, the effective use of rural tourism can contribute to the realization of rural revitalization.

3. To enhance the utilization of key resources and promote resource linkage, it is necessary to highlight the unique resources in the southwest mountainous areas of Anhui Province, which serve as a revolutionary base, possessing abundant red tourism resources. The red tourism resources can be integrated with regional culture to promote their joint development and linkage with other tourism resources while developing research and study tour products. In the southwest mountainous areas of Zhejiang Province, numerous traditional villages are distributed, but their influence is limited to Zhejiang Province. Therefore, it is important to implement a branding strategy and create a cross-regional traditional village tourism brand to generate brand effects. Additionally, historical and cultural protection and development should be reinforced, along with the protection of natural environments. It is essential to focus on the uniqueness of each village and avoid homogenized competition.

4. Attention should be given to the driving role of transportation and tourist source markets in rural tourism development, and tourism infrastructure construction should be strengthened. Areas with lagging rural tourism development often have relatively backward tourism infrastructure. Therefore, efforts should be made to strengthen the construction of tourism transportation facilities, catering and accommodations, and other supporting facilities to improve tourism reception capacity and enhance tourist satisfaction with the source market.

5. Promoting the deep integration of the primary and tertiary industries is crucial for rural tourism development. The ratio of the primary and tertiary industries to GDP is an important factor that affects rural tourism development. In fact, depending on the spatial location, the ratio can have positive or negative effects on rural tourism. Plain areas are suitable for agricultural development, where the primary industry has a higher GDP ratio. However, rural tourism development in these areas is not ideal. The key is to extend the agricultural industry chain, explore the multiple functions of agriculture, and transform from the primary industry of agricultural production and sales to the tertiary industry of agricultural tourism, study trips, and cultural creativity. In this process, agricultural technology research and development can also be integrated to carry out agricultural product processing and achieve the integration of the primary, secondary, and tertiary industries.

Furthermore, the paper also has several limitations. Rural tourism is a dynamic and continuously evolving sector, with the integration of modern tourism and rural industries becoming increasingly profound. To further deepen the study, additional influencing factors can be explored, and alternative analysis methods can be introduced to enhance the scientific validity of the conclusions. Moreover, the construction of NKRTVs as a national policy has received support from government departments at various levels, including provincial, municipal, and county governments. However, due to limitations in data availability, this paper has not yet quantified the statistical significance of policy factors. Future studies could address this gap by exploring and quantifying the effects of policy factors on rural tourism development.

## 6. Conclusions

This study focuses on the NKRTVs in the Yangtze River Delta region of China, where a comprehensive index system covering topographic conditions, land resources, tourism resources, cultural resources, socioeconomic status, and transport condition factors was constructed. Various spatial analysis methods and geographically weighted regression (GWR) models were integrated to investigate the spatial distribution pattern of NKRTVs in the Yangtze River Delta region and the spatial heterogeneity of their influencing factors. Based on the analysis, the following main conclusions can be drawn.

1. The distribution of NKRTVs in the Yangtze River Delta region is generally characterized by "small agglomeration, large dispersion". The main spatial agglomeration core is formed in Shanghai, while a secondary agglomeration core is formed in Huangshan City, Anhui Province. The distribution of key villages in the other regions of Jiangsu, Zhejiang, and Anhui provinces is relatively scattered. NKRTVs exhibit significant spatial correlation in their distribution, and there are notable differences in the distribution of hot and cold spots among regions. The hot spot areas are located in Shanghai and the surrounding Taihu Lake urban agglomeration, while the cold spot areas are located in the northern plain areas of Anhui Province.

2. The correlation between the spatial distribution of NKRTVs and each influencing factor exhibits both positive and negative correlations, which vary depending on the spatial location. The performance difference is more pronounced in economically developed and less developed areas, as well as in plains and mountainous hilly areas. Specifically, arable land has a positive influence on the spatial distribution of NKRTVs in the mountainous areas of Zhejiang and a negative correlation in the plains of northern Anhui and Taihu Lake. The water body factor has a positive effect on rural tourism development in the plain areas of Hangzhou Bay and Taihu Lake, where there are many ancient water towns and a dense network of rivers. Additionally, tourist attractions and traditional villages generally exhibit a positive correlation with the distribution of NKRTVs. Notably, the distribution density of NKRTVs shows a negative correlation with GDP per capita in large cities such as Shanghai, Suzhou and Nanjing, while a positive correlation is shown in the rural areas around these large cities. The source market factor proves that in economically developed metropolitan areas, the closer to the urban source, the greater the distribution density of NKRTVs. The proportion of primary industry output value to GDP is positively correlated in the mountainous areas of southwest Zhejiang Province with the spatial distribution of NKRTVs and negatively correlated in the northern plains with a single type of agriculture. The ratio of tertiary industry output to GDP is positively correlated with the spatial distribution of NKRTVs only in Shanghai and the surrounding areas of Shanghai.

## Supporting information

S1 Dataset (XLS)
